# Efficacy and safety of PD-1/PD-L1 inhibitor combined with chemotherapy versus chemotherapy alone in the treatment of advanced gastric or gastroesophageal junction adenocarcinoma: a systematic review and meta-analysis

**DOI:** 10.3389/fonc.2023.1077675

**Published:** 2023-04-11

**Authors:** Bo-Wei Liu, Qi-Xing Shang, Yu-Shang Yang, Long-Qi Chen

**Affiliations:** Department of Thoracic Surgery, West China Hospital of Sichuan University, Chengdu, Sichuan, China

**Keywords:** PD- 1/L1, chemotherapy, gastroesophageal (GEJ) adenocarcinoma, immunotherapy, PD-L 1, esophageal adenocarcinoma (EAC)

## Abstract

**Background:**

There is increasing evidence that immunotherapy (programmed cell death-1 (PD-1) inhibitor) combined with chemotherapy is superior to chemotherapy alone in neoadjuvant therapy for patients with previously untreated, unresectable advanced, or metastatic esophageal adenocarcinoma (EAC)/gastric/gastroesophageal junction adenocarcinoma (GEA). However, the results of recent studies have been contradictory. Therefore, the aim of this article is to evaluate the efficacy and safety of PD-1 inhibitors combined with chemotherapy in neoadjuvant therapy through meta-analysis.

**Method:**

We comprehensively reviewed the literature and clinical randomized controlled trials (RCTs) by February 2022 by searching Medical Subject Headings (MeSH) and keywords such as “esophageal adenocarcinoma” or “immunotherapy” in several databases, including the Embase, Cochrane, PubMed, and ClinicalTrials.gov websites. Two authors independently selected studies, extracted data, and assessed the risk of bias and quality of evidence by using standardized Cochrane Methods procedures. The primary outcomes were 1-year overall survival (OS) and 1-year progression-free survival (PFS), estimated by calculating the 95% confidence interval (CI) for the combined odds ratio (OR) and hazard ratio (HR). Secondary outcomes estimated using OR were disease objective response rate (DORR) and incidence of adverse events.

**Results:**

Four RCTs with a total of 3,013 patients researching the efficacy of immunotherapy plus chemotherapy versus chemotherapy alone on gastrointestinal cancer were included in this meta-analysis. The results showed that immune checkpoint inhibitor plus chemotherapy treatment was associated with an increased risk of PFS (HR = 0.76 [95% CI: 0.70–0.83]; p < 0.001), OS (HR = 0.81 [95% CI: 0.74–0.89]; p < 0.001), and DORR (relative ratio (RR) = 1.31 [95% CI: 1.19–1.44]; p < 0.0001) when compared with chemotherapy alone in advanced, unresectable, and metastatic EAC/GEA. However, immunotherapy combined with chemotherapy increased the incidence of adverse reactions such as alanine aminotransferase elevation (OR = 1.55 [95% CI: 1.17–2.07]; p = 0.003) and palmar-plantar erythrodysesthesia (PPE) syndrome (OR = 1.30 [95% CI: 1.05–1.63]; p = 0.02). Nausea (OR = 1.24 [95% CI: 1.07–1.44]; p = 0.005) and white blood cell count decreased (OR = 1.40 [95% CI: 1.13–1.73]; p = 0.002), and so on. Fortunately, toxicities were within acceptable limits. Meanwhile, for patients with a combined positive score (CPS) ≥1, compared with chemotherapy alone, immunotherapy combined with chemotherapy had a better overall survival rate (HR = 0.81 [95% CI: 0.73–0.90]; p = 0.0001).

**Conclusion:**

Our study shows that immunotherapy plus chemotherapy has an obvious benefit for patients with previously untreated, unresectable advanced, or metastatic EAC/GEA when compared with chemotherapy alone. However, a high risk of adverse reactions may occur during immunotherapy plus chemotherapy, and more studies focusing on the treatment strategies of untreated, unresectable advanced, or metastatic EAC/GEA are warranted.

**Systematic review registration:**

www.crd.york.ac.uk, identifier CRD42022319434.

## 1 Introduction

Esophageal cancer has become the sixth leading cause of cancer death, with over 500,000 deaths (5.5%) annually (1). Surgery has been considered the mainstream treatment for esophageal cancer for nearly half a century. The emergence of new technologies and deepening research on new cancer targets have led to markedly reduced operative mortality for patients (2). It is worth noting that the incidence of gastroesophageal junction adenocarcinoma (GEA) has gradually increased with the decline in the incidence of esophageal squamous cell carcinoma in recent years, especially in some European countries and the United States (3). Studies have shown that esophageal adenocarcinoma (EAC) and GEA have similar molecular profiles and clinical outcomes to systemic chemotherapy in advanced settings, so we have reason to believe that the treatment modalities of EAC/GEA are similar. Since there is still no standard classification system for GEA, the widely used Siewert classification is based on the location of the tumor center under the inner diameter to classify GEA into three types (4). However, some gastroesophageal junction tumors usually cross the full length of these parts in the esophagus, which makes it difficult to distinguish the center of the tumor under endoscopy, resulting in limitations in diagnosis and treatment. Although the current treatment strategy for GEA is still based on surgical resection, the 5-year survival rate of patients is approximately 30% due to the high rate of distant metastasis and local recurrence (5). Therefore, to improve the surgical resection rate and the long-term survival rate of patients, multimodal strategies including radiotherapy, chemotherapy, and other neoadjuvant and adjuvant therapies are gradually developing. A randomized controlled study showed that perioperative epirubicin, cisplatin, and infused fluorouracil (ECF) combined with surgery was associated with improved overall survival when compared with surgery alone (overall survival; 5-year overall survival (OS) rate of 36% *vs.* 23%; hazard ratio (HR) = 0.75 [95% confidence interval (CI): 0.60–0.93]; p = 0.02) (6). The FLOT4-AIO trial also demonstrated significant superiority when using the perioperative chemotherapy regimen FLOT, including platinum, docetaxel, and other drugs, which can improve the overall survival rate and median disease-free survival rate of patients, tumor shrinkage, and R0 resection rate (7). The CROSS study, based on the comparison of the efficacy of neoadjuvant chemoradiotherapy and surgery alone, showed that preoperative chemoradiotherapy could reduce the risk of regional disease progression (22% *vs.* 38%) and distant disease progression (39% *vs.* 48%) and improve the median overall survival (49 months *vs.* 24 months), 2- and 5-year OS (67% *vs.* 50%; [47% *vs.* 34%], HR = 0.665), and R0 resection rate (92% *vs.* 69%, p < 0.001) (8). Meanwhile, a meta-analysis showed that either neoadjuvant chemoradiotherapy or neoadjuvant chemotherapy significantly improved 2-year survival when compared with surgery alone (HR = 0.81 [95% CI: 0.70–0.93]; p = 0.002; and HR = 0.90 [95% CI: 0.81–1.00]; p = 0.05, respectively) (9). These findings provide an evidence-based framework for the formulation of therapeutic strategies for gastroesophageal junction adenocarcinoma. However, for unresectable advanced or metastatic gastroesophageal adenocarcinoma, the median OS is less than 12 months with fluoropyrimidine- or platinum-based chemotherapy regimens (2, 10–13).

In recent years, immunotherapy has gradually provided new ideas for the treatment of esophageal cancer. Programmed cell death-1 (PD-1) is widely expressed in various immune cells, such as CD4 T cells and CD8 T cells. Its main role is to restrict the activity of autoimmunity and autoimmune T cells during infection. PD-1 on tumor micro-infiltrating cells upregulates the proliferation and activation of T-regulatory cells by binding to programmed death ligand-1 (PD-L1) antibody expressed on tumor cells, thereby evading immune surveillance and evading tumor cell killing (14, 15). Therefore, immunosuppressive agents targeting the PD-1 pathway provide a new direction for cancer treatment. In addition, anti-PD-1 antibodies have considerable efficacy in the treatment of cancers such as small cell lung cancer and melanoma (16). The ATTRACTION-2 trial showed that the PD-1 inhibitor nivolumab significantly improved median OS compared to placebo for patients with advanced or recurrent gastric or GEA cancer (5.26 vs 4.14 months; p<0.0001), and the 12-month OS rate and progression-free survival (PFS) rate were 26.2% *vs.* 10.9% and 7.6% *vs.* 1.5%, respectively (17). At the same time, pembrolizumab has also been demonstrated to have antitumor efficacy and safety in the KEYNOTE-059 study (18). Some studies have reported that chemotherapeutic agents not only act through their cytotoxicity but also modulate the immune system and inhibit immunosuppressive cells, activating immune effector cells to promote the antitumor immune response (19, 20). Although immunotherapy has promising advantages in the treatment of locally advanced esophageal cancer, immune-related side effects, such as leukopenia, anemia, rash, fatigue, and diarrhea, cannot be ignored. Therefore, the safety of immunotherapy combined with chemotherapy compared with chemotherapy alone is worthy of evaluation and comparison. Hence, it is advisable to explore whether chemotherapy combined with PD-1 inhibitors would significantly improve the prognosis of patients with unresectable advanced or metastatic gastroesophageal adenocarcinoma, as safety evaluation is valuable.

Although there are several trials that have analyzed the efficacy and superiority of PD-1 inhibitors combined with chemotherapy in treating patients who had previously advanced, unresectable, and metastatic EAC/GEA, when compared to chemotherapy alone, the results are contradictory (21, 22). It is necessary to conduct a pooled analysis to assess whether PD-1 inhibitors combined with chemotherapy have a significant advantage over chemotherapy alone. Thus, we integrated and summarized the data of published studies, compared the efficacy and safety of immunotherapy combined with chemotherapy and chemotherapy alone, and comprehensively analyzed and drew conclusions to provide an evidence base for the treatment of previously untreated, unresectable advanced, or metastatic gastroesophageal adenocarcinoma.

## 2 Method

### 2.1 Literature

This study was performed according to the Preferred Reporting Items for Systematic Reviews and Meta-Analyses (PRISMA) statement. We searched for identified randomized controlled trials (RCTs) published before 31 December 2021 through electronic databases including PubMed, Embase, Cochrane, and ClinicalTrials.gov (https://clinicaltrials.gov). We identified articles by using the Medical Subject Headings and Test-word search strategy. Keywords included “neoadjuvant chemotherapy” or “esophageal adenocarcinoma”, “gastro-esophageal adenocarcinoma”, “PD-1”, and “randomized trial”, combined with AND/OR. The specific search strategy was attached in additional files. The eligibility criteria of patients were as follows: the studies included patients with unresectable, untreated (if prior therapy was more than 180 days), and metastatic EAC/GEA. Comparisons between chemotherapy combined with PD-1 or PD-L1 inhibitors and chemotherapy alone (either FLOT or other standard regimens) were performed. Studies were included only if the main outcomes on PFS, OS, or both and adverse events were reported, and the search was restricted to the English language only. Both randomized phase 2 and phase 3 trials were included. Hazard ratio (HR) and odds ratio (OR) for antitumor activity, survival outcomes, and safety indicators were available. The exclusion criteria were as follows: studies that were not RCTs and studies in which too many patients were lost to follow-up or follow-up data were incomplete or no valid information was available.

### 2.2 Data extraction

Data were extracted and reviewed by two independent researchers (Shang and Liu). Discrepancies were resolved by a third researcher (Yang). HR and 95% CIs of treatment with PD-1/PD-L1 inhibitors combined with chemotherapy compared to standard treatment with chemotherapy alone on PFS and OS were obtained. Relative ratio (RR) was used to evaluate the disease objective response rate (DORR). OS was selected as the primary endpoint, while PFS and DORR were secondary endpoints. Adverse event (AE) was the main measure of the safety profile. Predisposed subgroups are mainly focused on the combined positive score (CPS) (<1 *vs.* >1). All data were extracted from primary publications and their appendix.

### 2.3 Quality assessment

The Cochrane Risk of Bias Tool (23) was used to assess the risk of bias in RCT studies, including the following factors: random sequence generation, allocation concealment, blinding of participants and personnel, blinding of outcome assessment, incomplete outcome data, and selective reporting.

### 2.4 Statistical analysis

Each HR and CI collected from independent research was pooled into a meta-analysis by using the Mantel-Haenszel and random-effects models. The same method was used in the subgroup analysis. Heterogeneity was evaluated using Q and I^2^ statistics. Statistically significant heterogeneity was defined as a Cochran p < 0.1 or I^2^ greater than 50% (24). Sensitivity analyses were not conducted because of the limited number of included studies. All statistical analyses were performed using RevMan version 5.4 software and EndNote X9 version and the R programming language (version 3.6.3, Vienna, Austria).

## 3 Results

**Figure 1:**
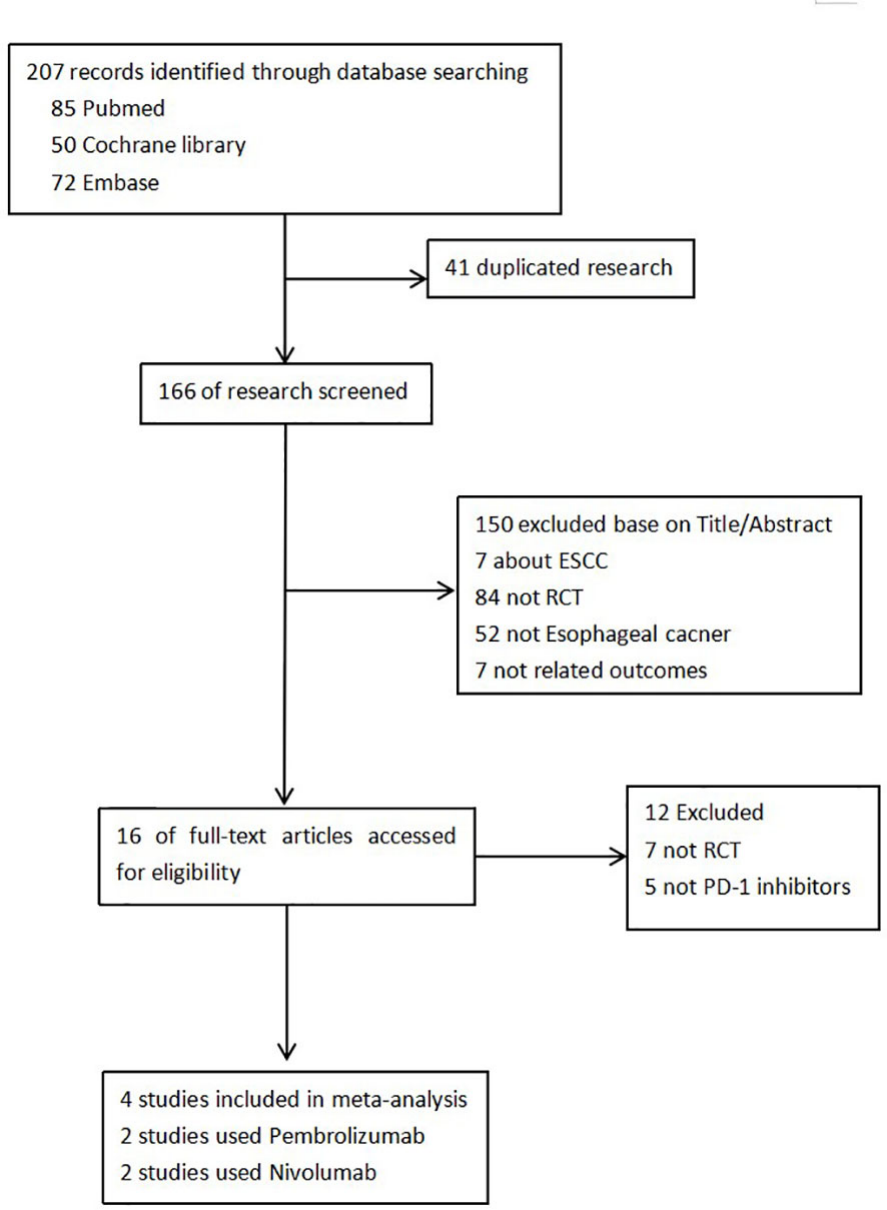
Flowchart of study selection.

**Figure 2:**
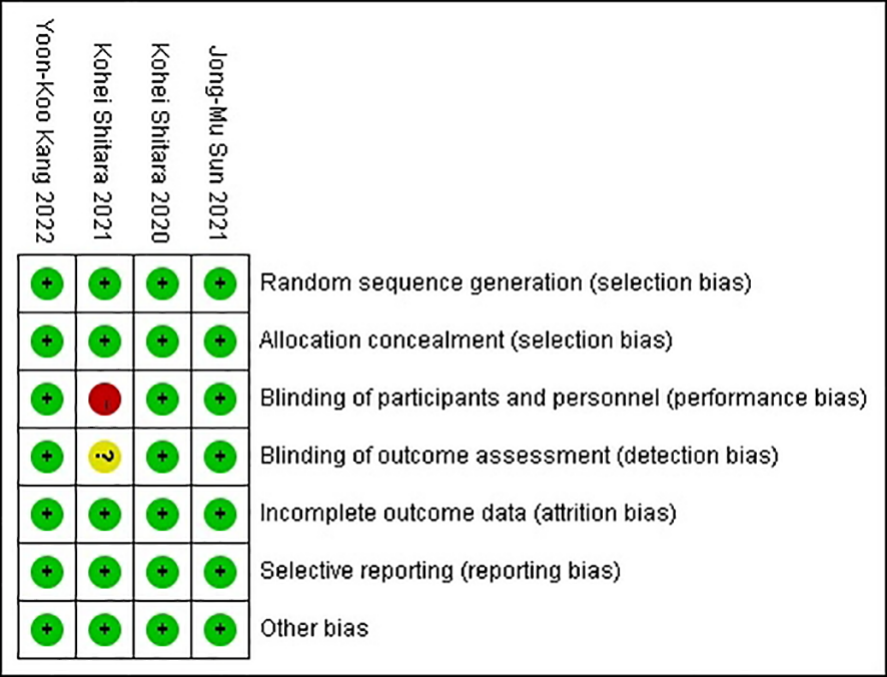
Quality assessment result of included studies according to the Cochrane risk of bias tool.

We retrieved 207 records in total. Four RCTs were finally included after screening according to the inclusion and exclusion criteria (21, 22, 25, 26). **Figure 1** shows the selection process. There were 3,013 patients included in our study. The basic characteristics of each study and quality assessment are shown in **Table 1** and **Figure 2**. In KEYNOTE-059, advanced, unresectable, and metastatic EAC/GEA patients were only analyzed in the subgroup, but information on OS and PFS can be accessed in its accessories. Pembrolizumab and nivolumab were regarded as the main immune checkpoint inhibitors (ICIs) in the trials.

**Table 1:** Basic characteristics of the included studies.

Study (year)	Design	Arms	Country	Population (N, age)	Sex (male/female)	Primary endpoint	Main outcome measures
Janjigian et al. (21)	RCT; multicenter; open-label; phase 3 study	Nivolumab plus chemotherapy (XELOX every 3 weeks or FOLFOX every 2 weeks) or chemotherapy alone	USA	N = 1,581, NM	1,100/481	OS/PFS	OS; PFS; ORR; AE
Jong-Mu Sun et al.	RCT; multicenter; double-blind, phase 3 study	Pembrolizumab plus chemotherapy (5-fluorouracil plus cisplatin every 3 weeks) or chemotherapy alone	USA	N = 201, NM	NM	OS/PFS	OS; PFS; ORR; DORR; AE
Yoon-Koo Kang et al. (21)	RCT; multicenter; double-blind, phase 3 study	Nivolumab plus chemotherapy (oxaliplatin plus S-1 or capecitabine every 3 weeks) or chemotherapy alone	Japan	N = 724, 25–89	523/201	OS/PFS	OS; PFS; ORR; DORR; DCR; AE
Kohei Shitara et al. (25)	RCT; partially blinded; phase 3 study	Pembrolizumab plus chemotherapy (cisplatin plus fluorouracil or capecitabine every 3 weeks) or chemotherapy alone	USA	N = 507, 22–87	374/133	OS/PFS	OS; ORR; DORR; PFS; AE

DORR, disease objective response rate; ORR, objective response rate; AE, adverse event; PFS, progression-free survival; OS, overall survival; NM, not mentioned; RCT, randomized control trial; DCR, disease control rate; XELOX, oxaliplatin plus capecitabine; FOLFOX, fluorouracil plus oxaliplatin.

### 3.1 Overall survival

**Figure 3:**
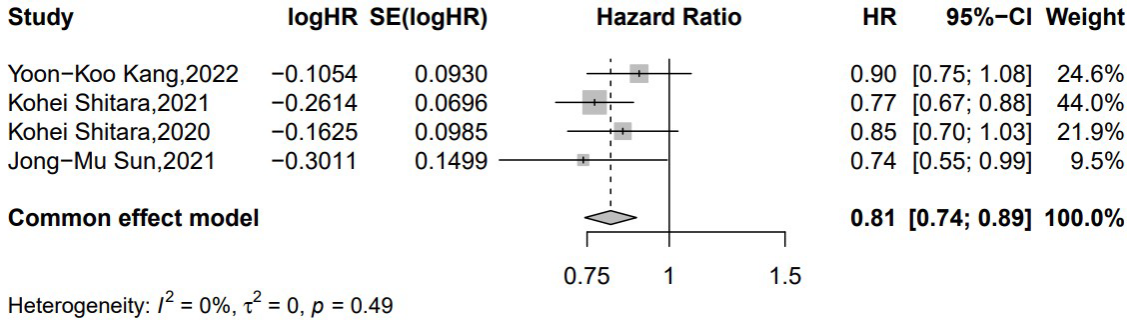
Forest plot of the hazard ratio comparing the overall survival (OS) in patients treated with immune checkpoint inhibitors (ICIs) + chemotherapy and chemotherapy alone.

Four studies reported OS (21, 22, 25, 26); 356 patients and 1,151 patients received pembrolizumab- and nivolumab-based chemotherapy, respectively. There was no significant heterogeneity among the studies. The results show that ICIs plus chemotherapy improved the median OS compared to standard chemotherapy in the first or subsequent treatment of advanced EAC/GEA. This is similar to the significance suggested by our pooled (HR = 0.81 [95% CI: 0.74–0.89]; p < 0.001) without obvious heterogeneity (p = 0.49, I^2^ = 0%) (**Figure 3**).

### 3.2 Progression-free survival

**Figure 4:**
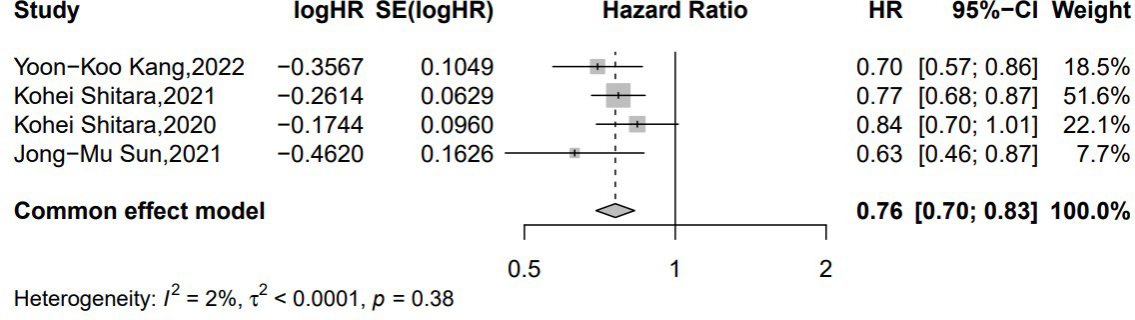
Forest plot of the hazard ratio comparing the progression-free survival (PFS) in patients treated with immune checkpoint inhibitors (ICIs) + chemotherapy and chemotherapy alone.

Four studies provided the median PFS (21, 22, 25, 26). In the common-effects model, there was no obvious heterogeneity (p = 0.38, I^2^ = 0%) within the included studies. ICIs plus chemotherapy in advanced, unresectable, and metastatic EAC/GEA significantly improved the patients’ median PFS when compared to chemotherapy alone (HR = 0.76 [95% CI: 0.70–0.83]; p < 0.001) (**Figure 4**).

### 3.3 Disease objective response rate

**Figure 5:**
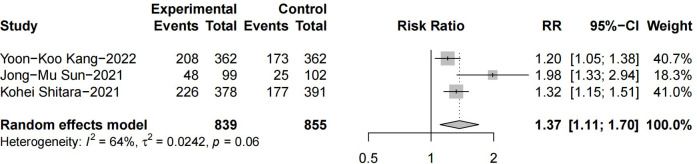
Forest plot of the hazard ratio comparing the disease objective response rate (DORR) in patients treated with immune checkpoint inhibitors (ICIs) + chemotherapy and chemotherapy alone.

Regarding the disease response rate, our results demonstrated that patients benefited from ICIs plus chemotherapy when compared to chemotherapy alone (RR = 1.37 [95% CI: 1.11–1.70]; p = 0.004; **Figure 5**), and there was no conspicuous heterogeneity (p = 0.06, I^2^ = 64%) within these studies.

### 3.4 Adverse event rate

After a comprehensive analysis of the data from the included studies, we found that ICIs combined with chemotherapy resulted in more treatment-related adverse events (TRAEs) than chemotherapy alone. The statistically significant characteristics were increased alanine aminotransferase (OR = 1.55 [95% CI: 1.17–2.07]; p = 0.003), increased aspartate aminotransferase (OR = 1.63 [95% CI: 1.27–2.10]; p = 0.0001), diarrhea (OR = 1.22 [95% CI: 1.03–1.43]; p = 0.02), nausea (OR = 1.24 [95% CI: 1.07–1.44]; p = 0.005), etc. (**Table 2**).

**Table 2:** Pooled results of adverse effects in patients during treatment.

Variables	No. of subjects	OR (95% CI)	p-Value
ALA increased	216	1.55 (1.17–2.07)	**0.003**
Anemia	678	1.16 (0.98–1.38)	0.09
ASA increased	289	1.63 (1.17–2.10)	**<0.000**
Decreased appetite	828	1.01 (0.86–1.19)	0.89
Diarrhea	819	1.22 (1.03–1.43)	**0.02**
Fatigue	702	1.08 (0.91–1.28)	0.37
Nausea	1,268	1.24 (1.07–1.44)	**0.005**
Neutropenia	594	0.99 (0.82–1.19)	0.9
NCD	661	1.35 (1.14–1.61)	**<0.000**
Peripheral neuropathy	550	1.21 (1.00–1.46)	**0.05**
PSN	691	1.18 (1.00–1.41)	0.06
PLT count decreased	617	1.14 (0.96–1.37)	0.14
PPE syndrome	367	1.30 (1.05–1.63)	**0.02**
Pruritus	88	2.88 (1.74–4.76)	**<0.000**
Rash	96	3.23 (2.00–5.20)	**<0.000**
Thrombocytopenia	395	1.14 (0.92–1.41)	0.24
Vomiting	633	1.12 (0.94–1.34)	0.21
WBC count decreased	380	1.40 (1.13–1.73)	**0.002**

Statistically significant value (p < 0.05) favors ICIs + chemotherapy.

ALA, alanine aminotransferase; ASA, aspartate aminotransferase; PCD, neutrophil count decreased; PLT, platelet; PSN, peripheral sensory neuropathy; PPE, palmar-plantar erythrodysesthesia syndrome; WBC, white blood cell; ICIs, immune checkpoint inhibitors.

Bold values represent that ICIs plus chemotherapy had a higher incidence of side effects and was statistically significant.

### 3.5 Subgroup analysis

**Figure 6:**
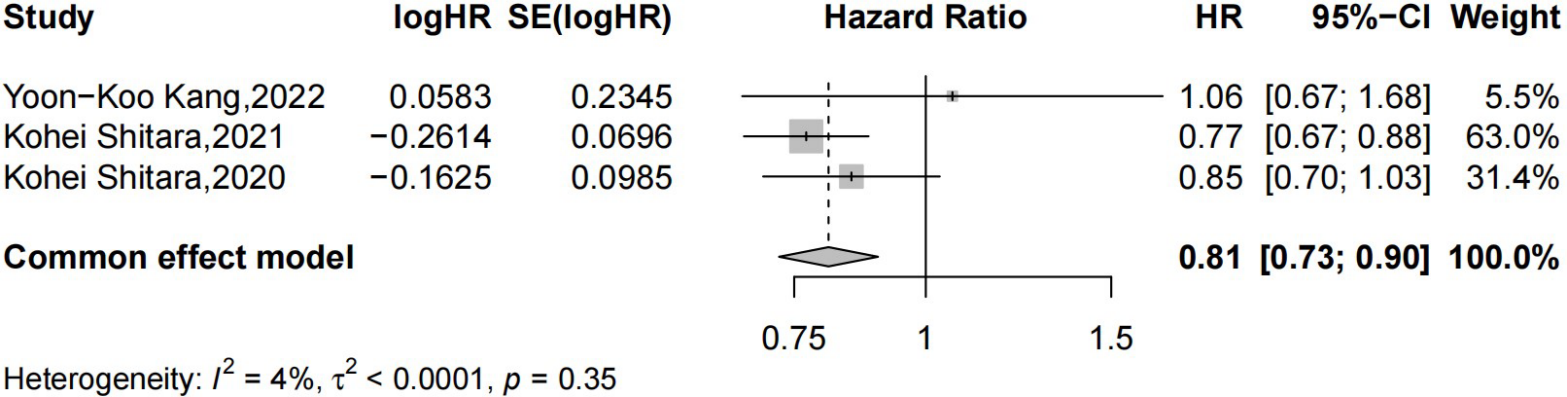
Forest plot of chemotherapy combined with PD-1/PD-L1 inhibitor versus chemotherapy alone in unresectable, untreated, or metastatic EAC/GEA patients with CPS ≥ 1. PD-1, programmed cell death-1; PD-L1, programmed death ligand-1; EAC, esophageal adenocarcinoma; GEA, gastroesophageal junction adenocarcinoma; CPS, combined positive score.

Three studies reported the median OS in CPS ≥ 1 (21, 22, 26). A total of 956 patients received ICIs plus chemotherapy, and 961 received chemotherapy alone. In our present random-effects model, the results from our meta-analysis suggest that for patients with CPS ≥ 1, ICIs + chemotherapy met the criteria for superiority compared with chemotherapy alone for overall survival (HR = 0.81 [95% CI: 0.73–0.90]; p = 0.0001) and without significant heterogeneity between studies (p = 0.35, I^2^ = 4%) (**Figure 6**).

## 4 Discussion

At present, there is controversy regarding the treatment of advanced, unresectable, and metastatic EAC/GEA (27). Although current platinum-based chemotherapy regimens have made some progress in improving the OS of patients with advanced, unresectable, and metastatic EAC/GEA, the 5-year survival rate of patients is still unsatisfactory. At the same time, with the gradual development of immunotherapy and its achievements in the treatment of esophageal cancer, some multicenter RCTs have been conducted to validate the value of ICIs + chemotherapy compared to chemotherapy alone. However, the results from different studies are contradictory. This study conducted a pooled analysis to investigate whether ICIs combined with chemotherapy have advantages over standard chemotherapy in the treatment of advanced, unresectable, and metastatic EAC/GEA. In some other malignant tumors, the effects of immunotherapy combined with chemotherapy versus chemotherapy alone are usually significant. In KEYNOTE-189, pembrolizumab plus pemetrexed–platinum significantly improved OS (HR = 0.56 [95% CI: 0.45–0.70]) and PFS (HR = 0.48 [95% CI: 0.40–0.58]) when compared with placebo plus pemetrexed–platinum in patients with metastatic non-small cell lung cancer (NSCLC) (28). The reason might be that when pemetrexed is co-administered with a PD-1 inhibitor, it can enhance immunosuppressive effects by recruiting or activating CD4/CD8 T cells and upregulating PD-L1 expression in advanced NSCLC through the activation of the NF-κB pathway (29).

In CheckMate-649, nivolumab plus chemotherapy treatment was associated with significant improvement in median overall survival (HR = 0.71 [98.4% CI: 0.59–0.86] p < 0.0001) when compared with chemotherapy in the PD-L1’s CPS of 5 and above population (21). However, patients did not achieve statistically superior OS with nivolumab plus chemotherapy when compared with chemotherapy alone in ATTRACTION-4 (HR = 0.90 [95% CI: 0.75–1.08]; p = 0.26) plus chemotherapy (22). The same results were also seen in KEYNOTE-062, which illustrated that pembrolizumab combined with chemotherapy had no clinical advantage in OS compared to chemotherapy alone (HR = 0.85 [95% CI: 0.70–1.03]; p = 0.05) (26).

The different populations, sample sizes, and types of immunosuppressants in different clinical trials also lead to uneven PFS in different trials. In the KEYNOTE-062 trial, nivolumab did not confer a sufficient benefit in terms of median progression-free survival (HR = 0.84 [95% CI: 0.70–1.02]; p = 0.04). However, CheckMate-649 showed that nivolumab conferred a PFS benefit in both CPS ≥ 1 and all randomized populations. Subgroup analysis also demonstrated the most significant improvement in PFS with CPS ≥ 5 (HR = 0.68 [98% CI: 0.56–0.81]; p < 0.0001. Pembrolizumab combined with chemotherapy in the treatment of advanced, unresectable, and metastatic EAC/GEA resulted in longer PFS (HR = 0.63 [98% CI 0.46–0.87]; p = 0.004) than chemotherapy alone in KEYNOTE-590. In a study from Asia, ATTRACTION-4, nivolumab plus chemotherapy was superior to chemotherapy alone in treating previously untreated, HER2-negative, unresectable, advanced, or recurrent gastric or gastroesophageal junction cancer by significantly improving the PFS (HR = 0.68 [98.5% CI: 0.51–0.90]; p = 0.0007) of patients, but it cannot translate into better overall survival. The ESCORT-1st trial also demonstrated that camrelizumab plus chemotherapy was associated with significantly improved OS (HR = 0.70 [95% CI: 0.56–0.88]; p = 0.01) and PFS (HR = 0.56 [95% CI: 0.46–0.68]; p < 0.001) (30). This is in contrast to CheckMate-648, in which although nivolumab plus chemotherapy compared with chemotherapy achieved a longer median PFS (6.8 *vs.* 4.3 months), the difference was not statistically significant (HR = 0.76 [95% CI: 0.56–1.03]) (31).

Therefore, to compare the true efficacy of ICIs plus chemotherapy with that of chemotherapy alone, we conducted this meta-analysis, and the results showed that when compared with chemotherapy alone, ICIs combined with chemotherapy can effectively improve the OS rate and PFS rate of all randomized patients or patients with CPS ≥ 1. According to the meta-analysis of the included studies, ICIs combined with chemotherapy did not significantly improve the DORR compared with placebo plus chemotherapy. Our research also shows a higher incidence of adverse events in ICIs plus chemotherapy than in chemotherapy alone. The reason could be that immunosuppressive agents increase the ratio of CD8+ and CD4+ T cells and tumor effector T cells (Teff), promote the production of inflammatory cytokines, and inhibit the activation of regulatory T cells and myeloid-derived suppressor cells, resulting in impaired self-tolerance (32).

The potential contributing factors for the differences within the included RCTs might be as follows: 1) due to the different sample sizes, the impact on the overall prognosis is different. Among them, it has been proven that in KEYNOTE-059, ICIs plus chemotherapy has a positive effect on the prognosis of patients. However, the sample size was too small to have a larger impact. 2) The proportions of patients who received subsequent therapy, including checkpoint inhibitors, were different. In ATTRACTION-4, CheckMate-649, and KEYNOTE-062 (66% *vs.* 39% *vs.* 50%), this discrepancy may be due to different medical practice modes, considering that patients in Asia are more likely to receive subsequent antitumor therapy than patients in Europe and the United States, which may lead to the difference in patient OS (10, 33). 3) The included studies have different chemotherapy regimens. Both ATTRACTION-4 and CheckMate-649 adopted oxaliplatin-based chemotherapy, whereas KEYNOTE-062 and KEYNOTE-590 used cisplatin-based chemotherapy, which resulted in different prognoses. In oxaliplatin-based chemotherapy studies (ATTRACTION-4 and CheckMate-649), the PFS of all randomized patients was (HR = 0.68 [98.51% CI: 0.51–0.90]); p = 0.0007) and (HR = 0.77 [95% CI: 0: 0.68–0.87]; p < 0.0001), respectively, when compared with chemotherapy alone. In the cisplatin-based KEYNOTE-062 trial, which included patients based on the eligibility criteria of CPS ≥ 1, the PFS of patients did not meet the criteria of superiority when compared with chemotherapy alone (6.9 *vs.* 6.4 months (HR = 0.84 [95% CI: 0.70–1.02]; p = 0.04). In addition, the proportions of 5-fluorouracil (5-FU) and its analogs in the ATTRACTION-4 and CheckMate-649 trials and KEYNOTE-590 and KEYNOTE-062 trials were 100%. *In vivo* research has proven that repeated cycles of 5-fluorouracil (the maximum dosage for the first cycle is three injections of 5-FU (40 mg/kg) and four injections per cycle for the following cycles) chemotherapy impaired antitumor immune functions, which may be another potential contributor to differences in treatment effects (34).

Apparently, our study has some limitations that should be addressed. The most important limitation was the fact that some of the studies we included were not classified according to the CPS score, which cannot provide adequate data. One study was a partially blinded RCT, which caused bias (26). An additional uncontrolled factor is that heterogeneity was a potential factor that may have affected the interpretation of the results. The source of heterogeneity in this study could be race, neoadjuvant chemoradiotherapy (nCRT) regimen, cutoff value, and ypTNM stage.

In fact, through our pooled analysis of the treatment of advanced, unresectable, and metastatic GEA, immunotherapy combined with chemotherapy will significantly improve the survival rate of patients while bringing more adverse effects, which will inevitably bring more restrictions to the treatment. Fortunately, the associated side effects were manageable and of low severity and therefore not of serious consequence. However, some studies have proven that chemotherapy patients can benefit more from immunotherapy. Therefore, the method to treat such patients remains to be explored. At present, although currently available treatments for advanced, unresectable, and metastatic adenocarcinoma of the esophagogastric junction are still limited, the different combinations of treatments might bring unpredictable outcomes. Meanwhile, with the application of particle therapy, there will be more options and possibilities for the treatment of esophageal cancer in the future.

## 5 Conclusion

In conclusion, ICIs plus chemotherapy improved median OS, PFS, and ORR when compared with chemotherapy in patients with advanced, unresectable, and metastatic EAC/GEA, with manageable higher TRAEs. Therefore, the optimal timing, dose, and combination regimen of neoadjuvant ICI combined with chemotherapy in the treatment of esophageal cancer are worthy of further study.

## Data availability statement

The original contributions presented in the study are included in the article/supplementary material. Further inquiries can be directed to the corresponding author.

## Author contributions

B-WL, Q-XS and L-QC contributed to the design and implementation of the research, the analysis of the results, and the writing of the manuscript. All authors contributed to the article and approved the submitted version.

## References

[1] Sung H Ferlay J Siegel RL Laversanne M Soerjomataram I Jemal A . Global cancer statistics 2020: GLOBOCAN estimates of incidence and mortality worldwide for 36 cancers in 185 countries. CA Cancer J Clin (2021) 71(3):209–49. doi: 10.3322/caac.21660 33538338

[2] Ajani JA D'Amico TA Bentrem DJ Chao J Corvera C Das P . Esophageal and esophagogastric junction cancers, version 2.2019, NCCN clinical practice guidelines in oncology. J Natl Compr Canc Netw (2019) 17(7):855–83. doi: 10.6004/jnccn.2019.0033 31319389

[3] Torre LA Siegel RL Ward EM Jemal A . Global cancer incidence and mortality rates and trends–an update. Cancer Epidemiol Biomarkers Prev (2016) 25(1):16–27. doi: 10.1158/1055-9965.EPI-15-0578 26667886

[4] Rüdiger Siewert J Feith M Werner M Stein HJ . Adenocarcinoma of the esophagogastric junction: results of surgical therapy based on anatomical/topographic classification in 1,002 consecutive patients. Ann Surg (2000) 232(3):353–61. doi: 10.1097/00000658-200009000-00007 PMC1421149 10973385

[5] Greally M Agarwal R Ilson DH . Optimal management of gastroesophageal junction cancer. Cancer (2019) 125(12):1990–2001. doi: 10.1002/cncr.32066 30973648 PMC10172875

[6] Cunningham D Allum WH Stenning SP Thompson JN Van de Velde CJ Nicolson M . Perioperative chemotherapy versus surgery alone for resectable gastroesophageal cancer. N Engl J Med (2006) 355(1):11–20. doi: 10.1056/NEJMoa055531 16822992

[7] Al-Batran SE Homann N Pauligk C Goetze TO Meiler J Kasper S . Perioperative chemotherapy with fluorouracil plus leucovorin, oxaliplatin, and docetaxel versus fluorouracil or capecitabine plus cisplatin and epirubicin for locally advanced, resectable gastric or gastro-esophageal junction adenocarcinoma (FLOT4): a randomized, phase 2/3 trial. Lancet (2019) 393(10184):1948–57. doi: 10.1016/S0140-6736(18)32557-1 30982686

[8] Shapiro J van Lanschot JJB Hulshof MCCM van Hagen P van Berge Henegouwen MI . Neoadjuvant chemoradiotherapy plus surgery versus surgery alone for esophageal or junctional cancer (CROSS): long-term results of a randomized controlled trial. Lancet Oncol (2015) 16(9):1090–8. doi: 10.1016/S1470-2045(15)00040-6 26254683

[9] Gebski V Burmeister B Smithers BM Foo K Zalcberg J Simes J . Survival benefits from neoadjuvant chemoradiotherapy or chemotherapy in esophageal carcinoma: a meta-analysis. Lancet Oncol (2007) 8(3):226–34. doi: 10.1016/S1470-2045(07)70039-6 17329193

[10] Kang YK Kang WK Shin DB Chen J Xiong J Wang J . Capecitabine/cisplatin versus 5-fluorouracil/cisplatin as first-line therapy in patients with advanced gastric cancer: a randomized phase III noninferiority trial. Ann Oncol (2009) 20(4):666–73. doi: 10.1093/annonc/mdn717 19153121

[11] Ajani JA D'Amico TA Bentrem DJ Chao J Cooke D Corvera C . Gastric cancer, version 2.2022, NCCN clinical practice guidelines in oncology. J Natl Compr Canc Netw (2022) 20(2):167–92. doi: 10.6004/jnccn.2022.0008 35130500

[12] Park YH Lee JL Ryoo BY Ryu MH Yang SH Kim BS . Capecitabine in combination with oxaliplatin (XELOX) as a first-line therapy for advanced gastric cancer. Cancer Chemother Pharmacol (2008) 61(4):623–9. doi: 10.1007/s00280-007-0515-7 17522863

[13] Ajani JA Fodor MB Tjulandin SA Moiseyenko VM Chao Y Cabral Filho S . Phase II multi-institutional randomized trial of docetaxel plus cisplatin with or without fluorouracil in patients with untreated, advanced gastric, or gastroesophageal adenocarcinoma. J Clin Oncol (2005) 23(24):5660–7. doi: 10.1200/JCO.2005.17.376 16110025

[14] Han Y Liu D Li L . PD-1/PD-L1 pathway: current studies in cancer. Am J Cancer Res (2020) 10(3):727–42. PMC7136921 32266087

[15] Baba Y Nomoto D Okadome K Ishimoto T Iwatsuki M Miyamoto Y . Tumor immune microenvironment and immune checkpoint inhibitors in esophageal squamous cell carcinoma. Cancer Sci (2020) 111(9):3132–41. doi: 10.1111/cas.14541 PMC7469863 32579769

[16] Xia L Liu Y Wang Y . PD-1/PD-L1 blockade therapy in advanced non-small cell lung cancer: Current status and future directions. Oncologist (2019) 24(Suppl 1):S31–41. doi: 10.1634/theoncologist.2019-IO-S1-s05 PMC6394772 30819829

[17] Kang YK Boku N Satoh T Ryu MH Chao Y Kato K . Nivolumab in patients with advanced gastric or gastro-esophageal junction cancer refractory to, or intolerant of, at least two previous chemotherapy regimens (ONO-4538-12, ATTRACTION-2): a randomized, double-blind, placebo-controlled, phase 3 trial. Lancet (2017) 390(10111):2461–71. doi: 10.1016/S0140-6736(17)31827-5 28993052

[18] Fuchs CS Doi T Jang RW Muro K Satoh T Machado M . Safety and efficacy of pembrolizumab monotherapy in patients with previously treated advanced gastric and gastroesophageal junction cancer: Phase 2 clinical KEYNOTE-059 trial. JAMA Oncol (2018) 4(5):e180013. doi: 10.1001/jamaoncol.2018.0013 29543932 PMC5885175

[19] Heinhuis KM Ros W Kok M Steeghs N Beijnen JH Schellens JHM . Enhancing antitumor response by combining immune checkpoint inhibitors with chemotherapy in solid tumors. Ann Oncol (2019) 30(2):219–35. doi: 10.1093/annonc/mdy551 30608567

[20] Wang W Wu L Zhang J Wu H Han E Guo Q . Chemoimmunotherapy by combining oxaliplatin with immune checkpoint blockades reduced tumor burden in colorectal cancer animal model. Biochem Biophys Res Commun (2017) 487(1):1–7. doi: 10.1016/j.bbrc.2016.12.180 28042031

[21] Janjigian YY Shitara K Moehler M Garrido M Salman P Shen L . First-line nivolumab plus chemotherapy versus chemotherapy alone for advanced gastric, gastro-esophageal junction, and esophageal adenocarcinoma (CheckMate 649): a randomized, open-label, phase 3 trial. Lancet (2021) 398(10294):27–40. doi: 10.1016/S0140-6736(21)00797-2 34102137 PMC8436782

[22] Kang YK Chen LT Ryu MH Oh DY Oh SC Chung HC . Nivolumab plus chemotherapy versus placebo plus chemotherapy in patients with HER2-nGEAtive, untreated, unresectable advanced or recurrent gastric or gastro-esophageal junction cancer (ATTRACTION-4): a randomized, multicenter, double-blind, placebo-controlled, phase 3 trial. Lancet Oncol (2022) 23(2):234–47. doi: 10.1016/S1470-2045(21)00692-6 35030335

[23] Higgins JP Altman DG Gøtzsche PC Jüni P Moher D Oxman AD . Cochrane bias methods group; cochrane statistical methods group. the cochrane collaboration's tool for assessing risk of bias in randomized trials. BMJ (2011) 343:d5928. doi: 10.1136/bmj.d5928 22008217 PMC3196245

[24] Higgins JP Thompson SG Deeks JJ Altman DG . Measuring inconsistency in meta-analyses. BMJ (2003) 327(7414):557–60. doi: 10.1136/bmj.327.7414.557 PMC192859 12958120

[25] Sun JM Shen L Shah MA Enzinger P Adenis A Doi T . KEYNOTE-590 investigators. pembrolizumab plus chemotherapy versus chemotherapy alone for first-line treatment of advanced esophageal cancer (KEYNOTE-590): a randomized, placebo-controlled, phase 3 study. Lancet (2021) 398(10302):759–71. doi: 10.1136/bmj.327.7414.557 34454674

[26] Shitara K Van Cutsem E Bang YJ Fuchs C Wyrwicz L Lee KW . Efficacy and safety of pembrolizumab or pembrolizumab plus chemotherapy vs chemotherapy alone for patients with first-line, advanced gastric cancer: The KEYNOTE-062 phase 3 randomized clinical trial. JAMA Oncol (2020) 6(10):1571–80. doi: 10.1001/jamaoncol.2020.3370 PMC7489405 32880601

[27] Lin D Khan U Goetze TO Reizine N Goodman KA Shah MA . Gastroesophageal junction adenocarcinoma: Is there an optimal management? Am Soc Clin Oncol Educ Book (2019) 39:e88–95. doi: 10.1200/EDBK_236827 31099690

[28] Gadgeel S Rodríguez-Abreu D Speranza G Esteban E Felip E Dómine M . Updated analysis from KEYNOTE-189: Pembrolizumab or placebo plus pemetrexed and platinum for previously untreated metastatic nonsquamous non-small cell lung cancer. J Clin Oncol (2020) 38(14):1505–17. doi: 10.1200/JCO.19.03136 32150489

[29] Lu CS Lin CW Chang YH Chen HY Chung WC Lai WY . Antimetabolite pemetrexed primes a favorable tumor microenvironment for immune checkpoint blockade therapy. J Immunother Cancer (2020) 8(2):e001392. doi: 10.1136/jitc-2020-001392 33243934 PMC7692992

[30] Luo H Lu J Bai Y Mao T Wang J Fan Q . ESCORT-1st investigators. effect of camrelizumab vs placebo added to chemotherapy on survival and progression-free survival in patients with advanced or metastatic esophageal squamous cell carcinoma: The ESCORT-1st randomized clinical trial. JAMA (2021) 326(10):916–25. doi: 10.1001/jama.2021.12836 PMC8441593 34519801

[31] Kato K Doki Y Ogata T Motoyama S Kawakami H Ueno M . First-line nivolumab plus ipilimumab or chemotherapy versus chemotherapy alone in advanced esophageal squamous cell carcinoma: a Japanese subgroup analysis of open-label, phase 3 trial (CheckMate 648/ONO-4538-50). Esophagus (2022) 20(2):291–301. doi: 10.1007/s10388-022-00970-1 PMC10024660 36401133

[32] Kang JH Bluestone JA Young A . Predicting and preventing immune checkpoint inhibitor toxicity: Targeting cytokines. Trends Immunol (2021) 42(4):293–311. doi: 10.1016/j.it.2021.02.006 33714688

[33] Koizumi W Narahara H Hara T Takagane A Akiya T Takagi M . S-1 plus cisplatin versus s-1 alone for first-line treatment of advanced gastric cancer (SPIRITS trial): a phase III trial. Lancet Oncol (2008) 9(3):215–21. doi: 10.1016/S1470-2045(08)70035-4 18282805

[34] Wu Y Deng Z Wang H Ma W Zhou C Zhang S . Repeated cycles of 5-fluorouracil chemotherapy impaired antitumor functions of cytotoxic T cells in a CT26 tumor-bearing mouse model. BMC Immunol (2016) 17(1):29. doi: 10.1186/s12865-016-0167-7 27645787 PMC5028929

